# Supplementary material for: The Intrinsic and Instrumental Values of Blockchain to Provide Beef Traceability in Hong Kong, South Korea, and the United States
Source: Foods. 2023 Nov 22;12(23):4209. doi: 10.3390/foods12234209 (PMC10706415; doi:10.3390/foods12234209)
Supplement: Supplementary file 1 [file foods-12-04209-s001.zip › foods-2680472-supplementary.pdf]

Gallup 2020-164-011

Consumer Preferences for Blockchain Technology based Beef Cold Chain System

Hello,

This survey is a Consumer Preferences for Blockchain Technology based Beef Cold Chain System conducted by the Korea Maritime Institute, government-funded research institute.

It will be used as a reference material for government policies by researching beef consumer's opinion of blockchain technology, so it would be very much appreciated if you respond honestly.

Your participation in this survey is completely voluntary. So, if you feel uncomfortable answering any questions, you can withdraw from the survey at any point. It is very important for us to learn your opinions.

Your survey responses will be kept strictly confidential and data from survey will be used only for statistical purposes. The results will be reported in aggregate form, and your information will be coded and will remain confidential.

If you have questions at any time about the survey or the procedures, you may contact Jisung Jo at +82–51 –797 -4916 or jisungjo@kmi.re.kr

Thank you very much for your time and support.

GALLUP KOREA research Division VI
Sun Young Kim
Tel +82-2-3702-2102, Fax +82-2-3702-2628
E-mail sykim@gallup.co.kr

SQ1) What is your gender?

1. Male
2. Female

SQ2) Do you eat buy beef?

1. Yes.
2. No → If no, end survey and exclude from sample

SQ3) What is your age?

____________years old → If under age 18, end survey and exclude from sample

1. Over the past few years, has your consumption of beef steaks increased or decreased?

1. Increased → Go to Q2
2. Decreased → Go to Q3
3. Stayed the same → Go to Q4

<<show only for those to answered 1 to question 1>>

2. Why has your consumption of beef steaks increased? (check all that apply)

- ❑ My household income has changed
- ❑ The price of beef steaks has fallen
- ❑ My health status has changed
- ❑ Beef steaks have become tastier
- ❑ Beef steaks have become more convenient to cook
- ❑ Beef steaks' quality has improved
- ❑ Cattle welfare has improved
- ❑ Other meat options have become less attractive
- ❑ Beef has become safer to eat
- ❑ Other ____

<<show only for those to answered B to question 2>>

3. Why has your consumption of beef steaks decreased? (check all that apply)

- ❑ My household income has changed
- ❑ The price of beef steaks has increased
- ❑ My health status has changed
- ❑ Beef steaks have become less tasty
- ❑ Beef steaks have become less convenient to cook
- ❑ Beef steaks' quality has worsen
- ❑ Cattle welfare has fallen
- ❑ Other meat options have become more attractive
- ❑ Beef has become less safe to eat
- ❑ I more concerned about the environment
- ❑ Other ____

4. To what extent do you agree or disagree with the following questions?
<<randomize the order of these items; the last item is a “trap question”>>

	Strongly Disagree	Somewhat Disagree	Neither Agree nor Disagree	Somewhat Agree	Strongly Agree
Pork chops are tasty	❍	❍	❍	❍	❍
Beef steaks are tasty	❍	❍	❍	❍	❍
Chicken breasts are tasty	❍	❍	❍	❍	❍
Fish is tasty	❍	❍	❍	❍	❍
Pork chops are healthy	❍	❍	❍	❍	❍
Beef steaks are healthy	❍	❍	❍	❍	❍
Chicken breasts are healthy	❍	❍	❍	❍	❍
Fish is healthy	❍	❍	❍	❍	❍
Pork chops are affordable	❍	❍	❍	❍	❍
Beef steaks are affordable	❍	❍	❍	❍	❍
Chicken breasts are affordable	❍	❍	❍	❍	❍
Fish is affordable	❍	❍	❍	❍	❍
Pork chops are safe to eat	❍	❍	❍	❍	❍
Beef steaks are safe to eat	❍	❍	❍	❍	❍
Chicken breasts are safe to eat	❍	❍	❍	❍	❍
Fish is safe to eat	❍	❍	❍	❍	❍
Check somewhat disagree	❍	❍	❍	❍	❍

5. How often do you eat beef steaks?

- ❍ Once or less every Two months
- ❍ Once a Month
- ❍ 2-3 Times a Month
- ❍ Once a Week
- ❍ 2-5 Times a Week
- ❍ Daily

6. How often do you buy beef steaks?

- ❍ Never
- ❍ Once or less every Two months
- ❍ Once a Month
- ❍ 2-3 Times a Month
- ❍ Once a Week
- ❍ 2-5 Times a Week
- ❍ Daily

7. How important are the following items to you when deciding whether to buy beef steaks? Please choose the most important four items.

<<randomize the order of these items>>

- Naturalness (made without modern food technologies and ingredients)
- Taste (the flavor of the food in your mouth)
- Price (price you pay)
- Safety (eating the food will not make you sick)
- Convenience (how easy and fast the food is to cook and eat)
- Nutrition (amount and type of fat, proteins, vitamins, etc.)
- Novelty (the food is something new you haven't tried before)
- Origin (whether the food is grown locally, regionally, in the U.S. or overseas)
- Fairness (farmers, processors, retails and consumers equally benefit)
- Appearance (whether the food looks appealing and appetizing)
- Environmental Impact (effects of food production the environment)
- Animal Welfare (well-being of farm animals used in food production)
- Size (Thickness and area of the steak)

7-1. Please choose the least important four items.

<<randomize the order of these items>>

- Naturalness (made without modern food technologies and ingredients)
- Taste (the flavor of the food in your mouth)
- Price (price you pay)
- Safety (eating the food will not make you sick)
- Convenience (how easy and fast the food is to cook and eat)
- Nutrition (amount and type of fat, proteins, vitamins, etc.)
- Novelty (the food is something new you haven't tried before)
- Origin (whether the food is grown locally, regionally, in the U.S. or overseas)
- Fairness (farmers, processors, retails and consumers equally benefit)
- Appearance (whether the food looks appealing and appetizing)
- Environmental Impact (effects of food production the environment)
- Animal Welfare (well-being of farm animals used in food production)
- Size (Thickness and area of the steak)

8. What has been your household's usual WEEKLY expense for food bought during grocery shopping?

- ❍ less than $20
- ❍ $20 – $39
- ❍ $40 - $59
- ❍ $60 - $79
- ❍ $80 - $99
- ❍ $100 - $119
- ❍ $120 - $139
- ❍ $140 - $159
- ❍ $160 or more

9. What has been your household's usual WEEKLY expense for meals or snacks from restaurants, fast food places, cafeterias, carryout or other such places?

- ❍ less than $20
- ❍ $20 – $39
- ❍ $40 - $59
- ❍ $60 - $79
- ❍ $80 - $99
- ❍ $100 - $119
- ❍ $120 - $139
- ❍ $140 - $159
- ❍ $160 or more

10. Overall, how much do you know about each of the following topics?

<<randomize the order of these items>>

	Nothing	A little	A moderate amount	Quite a bit	A great deal
Blockchain technology	❍	❍	❍	❍	❍
HACCP	❍	❍	❍	❍	❍
Antibiotic use in livestock	❍	❍	❍	❍	❍
Genetically modified food	❍	❍	❍	❍	❍
Food imports	❍	❍	❍	❍	❍
CRISPR	❍	❍	❍	❍	❍
Lab grown meat	❍	❍	❍	❍	❍

Choice Experiment Questions

<<information statement>>

Now, imagine you are shopping at your local grocery store.

In what follows, we will ask you **10 different choice questions** that are all similar to each other; key differences include the prices charged for each option of beef steak and information such as the beef steak's supply chain traceability, traceability of temperature history, and traceability of country of origin. All options represent steaks of the same size, type, grade and weight.

For each question, we want to know **which steak option you would be most likely to buy**.

Traceability information is about all nodes of whole supply chain of beef from farm to consumer.
(farm → processing → import/export workshops → inland transportation → sea (or air) transportation → wholesale/retail companies → consumer)

Please answer as honestly as possible and in a manner that you think would truly reflect how you would actually shop.

<<information statement randomly provided to ½ of respondents>>

Some of the options below describe information about beef attributes that is provided by **blockchain technology**.
A blockchain is system of keeping a list of records (or “blocks”) and transmitting information in a way that is open (visible to everyone), resistant to manipulation (the information is truthful), and timely.
Thus, **it is useful to enhance the reliability and transparency of data** as it is virtually impossible to forge or alter it.
This technology is mainly used in financial transactions, and it is also being actively applied in the logistics sector for tracking and managing cargo. In the food sector, it is used for food history management and quality control.

(Show below statement before the first block of questions to only the respondents who do not receive the information statement above)

※ Even if you are not familiar with the Blockchain technology, **please choose your preferred option** based on the information provided in the table.

Block 1: (1/2 of respondents to be randomly assigned to this block of questions). The order of the choice questions should be randomized across respondents.

11. Which of the following would you choose?

	**Option A**	**Option B**	**Option C**	**Option D**
Price ($/lb)	$15.00	$7.00	$7.00	If these were the only options available, I would not buy beef
Supply Chain Traceability	No location traceable during all parts of the supply chain back to the farm	Location traceable during all parts of the supply chain back to the farm	No location traceable during all parts of the supply chain back to the farm	
Temperature History	Temperature exceeded safe levels for 20 minutes	No temperature history available	Temperature never exceeded safe levels	
Country of Origin	from Brazil (according to blockchain)	from Australia	from Canada (according to blockchain)	

12. Which of the following would you choose?

	**Option A**	**Option B**	**Option C**	**Option D**

Price ($/lb)	$7.00	$9.00	$6.00	If these were the only options available, I would not buy beef
Supply Chain Traceability	No location traceable during all parts of the supply chain back to the farm	Location traceable during all parts of the supply chain back to the farm (according to blockchain)	No location traceable during all parts of the supply chain back to the farm	
Temperature History	Temperature never exceeded safe levels (according to blockchain)	Temperature exceeded safe levels for 20 minutes	No temperature history available	
Country of Origin	from South Korea	from United States, (according to blockchain)	from Australia	

13. Which of the following would you choose?

	Option A	Option B	Option C	Option D
Price ($/lb)	$7.00	$8.00	$8.00	If these were the only options available, I would not buy beef
Supply Chain Traceability	No location traceable during all parts of the supply chain back to the farm	Location traceable during all parts of the supply chain back to the farm (according to blockchain)	Location traceable during all parts of the supply chain back to the farm	
Temperature History	Temperature exceeded safe levels for 20 minutes, (according to blockchain)	No temperature history available	Temperature never exceeded safe levels	
Country of Origin	from Canada	from South Korea, (according to blockchain)	from Brazil	

14. Which of the following would you choose?

	Option A	Option B	Option C	Option D
Price ($/lb)	$9.00	$7.00	$15.00	If these were the only options available, I would not buy beef
Supply Chain Traceability	Location traceable during all parts of the supply chain back to the farm (according to blockchain)	No location traceable during all parts of the supply chain back to the farm	No location traceable during all parts of the supply chain back to the farm	

Temperature History	Temperature exceeded safe levels for 20 minutes, (according to blockchain)	Temperature never exceeded safe levels	Temperature exceeded safe levels for 20 minutes
Country of Origin	from Canada	from Brazil, (according to blockchain)	from Canada

15. Which of the following would you choose?

	Option A	Option B	Option C	Option D
Price ($/lb)	$12.00	$13.00	$12.00	If these were the only options available, I would not buy beef
Supply Chain Traceability	No location traceable during all parts of the supply chain back to the farm	Location traceable during all parts of the supply chain back to the farm (according to blockchain)	Location traceable during all parts of the supply chain back to the farm	
Temperature History	No temperature history available	Temperature never exceeded safe levels	Temperature exceeded safe levels for 20 minutes, (according to blockchain)	
Country of Origin	from United States	from Australia, (according to blockchain)	from Canada	

16. Which of the following would you choose?

	Option A	Option B	Option C	Option D
Price ($/lb)	$10.00	$14.00	$5.00	If these were the only options available, I would not buy beef
Supply Chain Traceability	Location traceable during all parts of the supply chain back to the farm	No location traceable during all parts of the supply chain back to the farm	No location traceable during all parts of the supply chain back to the farm	
Temperature History	Temperature exceeded safe levels for 20 minutes, (according to blockchain)	Temperature exceeded safe levels for 20 minutes, (according to blockchain)	Temperature never exceeded safe levels	
Country of Origin	from South Korea, (according to blockchain)	from United States, (according to blockchain)	Origin unknown	

17. Which of the following would you choose?

	Option A	Option B	Option C	Option D
Price ($/lb)	$8.00	$5.00	$13.00	If these were the only options available, I would not buy beef
Supply Chain Traceability	Location traceable during all parts of the supply chain back to the farm	No location traceable during all parts of the supply chain back to the farm	Location traceable during all parts of the supply chain back to the farm (according to blockchain)	
Temperature History	No temperature history available	Temperature exceeded safe levels for 20 minutes	Temperature never exceeded safe levels, (according to blockchain)	
Country of Origin	from Canada, (according to blockchain)	from United States	from South Korea, (according to blockchain)	

18. Which of the following would you choose?

	Option A	Option B	Option C	Option D
Price ($/lb)	$9.00	$6.00	$10.00	If these were the only options available, I would not buy beef
Supply Chain Traceability	Location traceable during all parts of the supply chain back to the farm (according to blockchain)	No location traceable during all parts of the supply chain back to the farm	Location traceable during all parts of the supply chain back to the farm	
Temperature History	Temperature exceeded safe levels for 20 minutes	No temperature history available	Temperature never exceeded safe levels, (according to blockchain)	
Country of Origin	from South Korea, (according to blockchain)	from Canada	from Australia, (according to blockchain)	

19. Which of the following would you choose?

	Option A	Option B	Option C	Option D
Price ($/lb)	$6.00	$15.00	$7.00	If these were the only options available, I
Supply Chain Traceability	No location traceable during all parts of the supply chain back to the farm	No location traceable during all parts of the supply chain back to the farm	Location traceable during all parts of the supply chain back to the farm	

Temperature History	No temperature history available	Temperature exceeded safe levels for 20 minutes	Temperature exceeded safe levels for 20 minutes	would not buy beef
Country of Origin	from United States, (according to blockchain)	from Australia	from South Korea	

20. Which of the following would you choose?

	**Option A**	**Option B**	**Option C**	**Option D**
Price ($/lb)	$11.00	$6.00	$13.00	If these were the only options available, I would not buy beef
Supply Chain Traceability	Location traceable during all parts of the supply chain back to the farm (according to blockchain)	Location traceable during all parts of the supply chain back to the farm	Location traceable during all parts of the supply chain back to the farm (according to blockchain)	
Temperature History	Temperature never exceeded safe levels	Temperature exceeded safe levels for 20 minutes, (according to blockchain)	Temperature never exceeded safe levels, (according to blockchain)	
Country of Origin	from Australia	from Brazil	from United States, (according to blockchain)	

Block 2: (1/2 of respondents to be randomly assigned to this block of questions). The order of the choice questions should be randomized across respondents.

21. Which of the following would you choose?

	**Option A**	**Option B**	**Option C**	**Option D**
Price ($/lb)	$10.00	$11.00	$9.00	If these were the only options available, I would not buy beef
Supply Chain Traceability	Location traceable during all parts of the supply chain back to the farm	Location traceable during all parts of the supply chain back to the farm	Location traceable during all parts of the supply chain back to the farm (according to blockchain)	

Temperature History	Temperature never exceeded safe levels	No temperature history available	Temperature exceeded safe levels for 20 minutes, (according to blockchain)	
Country of Origin	from United States, (according to blockchain)	from South Korea	from Australia, (according to blockchain)	

22. Which of the following would you choose?

	Option A	Option B	Option C	Option D
Price ($/lb)	$13.00	$10.00	$9.00	If these were the only options available, I would not buy beef
Supply Chain Traceability	No location traceable during all parts of the supply chain back to the farm	No location traceable during all parts of the supply chain back to the farm	Location traceable during all parts of the supply chain back to the farm (according to blockchain)	
Temperature History	Temperature exceeded safe levels for 20 minutes, (according to blockchain)	Temperature never exceeded safe levels, (according to blockchain)	No temperature history available	
Country of Origin	from Canada, (according to blockchain)	from Australia, (according to blockchain)	from United States	

23. Which of the following would you choose?

	Option A	Option B	Option C	Option D
Price ($/lb)	$6.00	$11.00	$8.00	If these were the only options available, I would not buy beef
Supply Chain Traceability	Location traceable during all parts of the supply chain back to the farm (according to blockchain)	Location traceable during all parts of the supply chain back to the farm	No location traceable during all parts of the supply chain back to the farm	
Temperature History	Temperature exceeded safe levels for 20 minutes	Temperature never exceeded safe levels, (according to blockchain)	Temperature never exceeded safe levels, (according to blockchain)	

Country of Origin	from Brazil, (according to blockchain)	from United States, (according to blockchain)	from South Korea	

24. Which of the following would you choose?

	Option A	Option B	Option C	Option D
Price ($/lb)	$8.00	$8.00	$11.00	If these were the only options available, I would not buy beef
Supply Chain Traceability	Location traceable during all parts of the supply chain back to the farm (according to blockchain)	Location traceable during all parts of the supply chain back to the farm	No location traceable during all parts of the supply chain back to the farm	
Temperature History	Temperature never exceeded safe levels, (according to blockchain)	Temperature exceeded safe levels for 20 minutes	Temperature exceeded safe levels for 20 minutes, (according to blockchain)	
Country of Origin	from Brazil	from Canada, (according to blockchain)	from United States	

25. Which of the following would you choose?

	Option A	Option B	Option C	Option D
Price ($/lb)	$14.00	$5.00	$12.00	If these were the only options available, I would not buy beef
Supply Chain Traceability	Location traceable during all parts of the supply chain back to the farm	No location traceable during all parts of the supply chain back to the farm	No location traceable during all parts of the supply chain back to the farm	
Temperature History	Temperature never exceeded safe levels	Temperature exceeded safe levels for 20 minutes, (according to blockchain)	Temperature never exceeded safe levels	
Country of Origin	from Australia	Origin unknown	from Brazil	

26. Which of the following would you choose?

	Option A	Option B	Option C	Option D
Price ($/lb)	$12.00	$13.00	$14.00	

Supply Chain Traceability	No location traceable during all parts of the supply chain back to the farm	Location traceable during all parts of the supply chain back to the farm (according to blockchain)	Location traceable during all parts of the supply chain back to the farm	
Temperature History	No temperature history available	Temperature never exceeded safe levels, (according to blockchain)	Temperature exceeded safe levels for 20 minutes	If these were the only options available, I would not buy beef
Country of Origin	from South Korea, (according to blockchain)	from Brazil	from Australia	

27. Which of the following would you choose?

	Option A	Option B	Option C	Option D
Price ($/lb)	$5.00	$12.00	$11.00	
Supply Chain Traceability	No location traceable during all parts of the supply chain back to the farm	No location traceable during all parts of the supply chain back to the farm	Location traceable during all parts of the supply chain back to the farm (according to blockchain)	
Temperature History	Temperature never exceeded safe levels, (according to blockchain)	Temperature never exceeded safe levels	Temperature exceeded safe levels for 20 minutes	If these were the only options available, I would not buy beef
Country of Origin	Origin unknown	from South Korea, (according to blockchain)	from Canada	

28.Which of the following would you choose?

	Option A	Option B	Option C	Option D
Price ($/lb)	$13.00	$9.00	$6.00	
Supply Chain Traceability	No location traceable during all parts of the supply chain back to the farm	Location traceable during all parts of the supply chain back to the farm	No location traceable during all parts of the supply chain back to the farm	If these were the only options available, I would not buy beef
Temperature History	Temperature never exceeded safe levels, (according to blockchain)	Temperature never exceeded safe levels	Temperature exceeded safe levels for 20 minutes, (according to blockchain)	

Country of Origin	from Australia, (according to blockchain)	from Canada	from United States, (according to blockchain)	

29. Which of the following would you choose?

	Option A	Option B	Option C	Option D
Price ($/lb)	$5.00	$10.00	$10.00	If these were the only options available, I would not buy beef
Supply Chain Traceability	No location traceable during all parts of the supply chain back to the farm	Location traceable during all parts of the supply chain back to the farm (according to blockchain)	Location traceable during all parts of the supply chain back to the farm	
Temperature History	Temperature exceeded safe levels for 20 minutes	Temperature never exceeded safe levels, (according to blockchain)	No temperature history available	
Country of Origin	from Australia	from Canada	from South Korea, (according to blockchain)	

30. Which of the following would you choose?

	Option A	Option B	Option C	Option D
Price ($/lb)	$11.00	$12.00	$5.00	If these were the only options available, I would not buy beef
Supply Chain Traceability	Location traceable during all parts of the supply chain back to the farm (according to blockchain)	Location traceable during all parts of the supply chain back to the farm	No location traceable during all parts of the supply chain back to the farm	
Temperature History	Temperature never exceeded safe levels	Temperature exceeded safe levels for 20 minutes, (according to blockchain)	No temperature history available	
Country of Origin	from United States	from South Korea, (according to blockchain)	from Brazil, (according to blockchain)	

Demographic Questions

DQ1. Have you ever worked on a farm or ranch?

- ❍ Yes
- ❍ No

DQ2. What is your current marital status?

- ❍ Single, Never Married
- ❍ Married
- ❍ Separated
- ❍ Divorced
- ❍ Widowed

DQ3. What percentage of the grocery shopping do you do for your household?

- ❍ 100%
- ❍ 75-99%
- ❍ 50-74%
- ❍ 25-49%
- ❍ 1-25%
- ❍ 0%

DQ4. How many people (including yourself) live in your household?

- ❍ 1
- ❍ 2
- ❍ 3
- ❍ 4
- ❍ 5 or more

<<show only for those to answered **more than 2 people** to DQ4 >>
DQ4-1. Are there children under the age of 12 living in your household?

- ❍ Yes
- ❍ No

DQ5. In what US state do you live? <<Arrange 51 states from Alabama to Wyoming >>

▼ Alabama ... Wyoming

DQ6. What is the highest level of education you have completed?

- Less than High School
- High School/GED
- Some College
- 2-Year College Degree (Associates)
- 4-Year College Degree (BA, BS)
- Master's Degree
- Professional Degree (Ph.D., J.D., M.D., etc.)
